# Value of glycogen synthase 2 in intrahepatic cholangiocarcinoma prognosis assessment and its influence on the activity of cancer cells

**DOI:** 10.1080/21655979.2021.2005224

**Published:** 2021-12-07

**Authors:** Sigen A, Huijun Wu, Xiaodong Wang, Xidong Wang, Jiarui Yang, Long Xia, Yijun Xia

**Affiliations:** ^a^ Department of Hepatobiliary-Pancreatic-Splenic Surgery, Inner Mongolia Autonomous Region People’s Hospital, Hohhot, China; ^b^ Department of Hepatobiliary Surgery, The Third Affiliated Hospital, Sun Yat-sen University, Guangzhou, China

**Keywords:** Intrahepatic cholangiocarcinoma, glycogen synthase 2, prognosis, P53 signaling pathway

## Abstract

Intrahepatic cholangiocarcinoma (ICC) is the second most common primary liver tumor with increasing incidence worldwide. Metabolic reprogramming caused by metabolic related gene disorders is a prominent hallmark of tumors, among which Glycogen Synthase 2 (GYS2) is the key gene responsible for regulating cellular energy metabolism, and its expression disorders are closely related to various tumors and glycometabolic diseases. However, we still know nothing about its role in ICC. This study is intended to reveal the functional role of GYS2 in the ICC progress and explore the underlying mechanism. Based on the integrated pan-cancer analysis of GYS2 in the GEPIA database, the expression of GYS2 in paired ICC and adjacent non tumor tissues was detected by qPCR. It was found that the expression of GYS2 was significantly down-regulated in ICC. Further analysis showed that its low expression was not only associated with the degree of pathological differentiation, tumor size, microvascular invasion and lymph node metastasis, but also an independent risk factor for unfavorable prognosis. Functional studies have shown that GYS2 overexpression can significantly impair the proliferation, replication, cloning, migration and invasion of cholangiocarcinoma cells, while the silencing GYS2 dramatically promotes the development of the aforementioned phenotypes, the underlying mechanism may be that GYS2 activates the P53 pathway. In conclusions,low GYS2 expression in ICC predicted unfavorable patient outcomes; GYS2 overexpression could significantly impair the proliferation, migration and invasion of cholangiocarcinoma cells via activating the P53 pathway and GYS2 was expected to become a potential therapeutic target for such patients.

## Introduction

Intrahepatic cholangiocarcinoma (ICC) is a primary liver cancer with extremely malignant biological behavior second only to hepatocellular carcinoma (HCC) in incidence. In recent years, both the incidence and mortality have shown a significant increase trend. Due to the significant differences in biological characteristics from HCC and extrahepatic cholangiocarcinoma, it not only lacks characteristic clinical symptoms and tumor markers, but is also highly invasive, has a low surgical resection rate, and is prone to recurrence and metastasis after surgery, resulting in a very poor prognosis [1,2]. GEMOX regimen (Gemcitabine combined with oxaliplatin), has always been the first-line regimen for the treatment of advanced cholangiocarcinoma, but its Objective Response Rate (ORR) is only 15 ~ 26%. Drug resistance often occurs, and there is no standard second-line treatment [3]. It is urgent to improve the survival of patients with new treatment. In order to improve the clinical efficacy of ICC, it is necessary to study the molecular mechanism of ICC. Currently, some molecules such as FGFR have been found to be essential for ICC, and a few therapies targeting these molecules have been successfully applied to clinical practice and achieved good results [4,5]. However, because of the heterogeneity and complexity of ICC, more targets and new mechanisms are urgently needed.

Compared with normal cells, tumor cells have the potential of unlimited replication and proliferation, anti-apoptosis, sustained angiogenesis and the ability of invasion and metastasis, the energy supply and metabolic pathway supporting the above characteristics of tumor cells are the core of the study of tumor characteristics [6]. Therefore, in the regulation of energy metabolism, the involvement of rate-limiting enzyme or key enzyme is needed, especially the tumor-promoting effect of each enzyme should be paid enough attention [7,8]. Liver glycogen synthase, encoded by Glycogen Synthase-2 (GYS2), is a key rate-limiting enzyme that catalyzes glycogen synthesis. Its expression disorder is closely related to a variety of glucose metabolism diseases and tumors [9–12]. Current research has confirmed that expression of GYS2 was notably down-regulated in HCC and correlated with decreased glycogen content and unfavorable patient prognosis [11]. However, we are still in the dark about the role of GYS2 in ICC.

Given that GYS2 had significant liver expression characteristics [13], we proposed a scientific hypothesis that GYS2 was likely to play a key role in the occurrence and progression of ICC. Therefore, the aim of this study was to define the role of GYS2 in ICC and elucidate the underlying mechanism.

## Materials and methods

### Patients, tissue specimens and follow-up

A total of 56 pairs of ICC tissues and adjacent non-tumor tissues containing normal intrahepatic bile ducts) were collected from the Third Affiliated Hospital of Sun Yat-sen University for radical hepatectomy. The clinical specimens collected above had complete clinical and pathological data. All patients had not received radiotherapy, chemotherapy or interventional therapy before surgery and signed informed consent after admission. This study was approved by the Ethics Committee of the Third Affiliated Hospital of Sun Yat-sen University and the People’s Hospital of Inner Mongolia Autonomous Region, respectively. The prognosis information of the patients was obtained by outpatient, in-patient review or telephone follow-up.

### Bioinformatics analysis of data

GEPIA database [14] (Gene Expression Profiling Interactive Analysis,), a newly developed public database for cancer and normal gene expression profiling analysis, combines TCGA tumor big data (9,736 tumor RNA sequencing data) with GTEx normal tissue big data (8,587 normal samples of RNA sequencing data). It aims to use bioinformatics technology to solve important problems in cancer biology, such as revealing cancer subtypes, driver genes, alleles, differential expression or carcinogens, in order to delve into new drug intervention targets and biomarkers with clinical predictive value.

### Cell culture and transfection

Human ICC cell lines (QBC939 and HCCC9810) and normal human intrahepatic bile duct epithelial cells (HIBECs) were obtained from Cell Bank of the Chinese Academy of Sciences (Shanghai, China). They were maintained in Dulbecco modified Eagle medium (DMEM) (GIBCO, Rockville, MD, USA) with 10% fetal bovine serum (FBS) and cultured in 37°C incubator containing 5% CO_2_. The plasmid encoding GYS2 was cloned into the empty vector of recombinant plasmid pcDNA 3.1. According to the manufacturer’s proposal, lipofectamine 3000 (Invitrogen, Camarillo, CA, USA) was used for transfection. For the silencing of GYS2 gene, we commissioned RiboBio Co., Ltd. (Guangzhou, China) to design and construct the siRNA for GYS2, and its sequence was as follows:

siGYS2 #1, 5ʹ-ACCAATAAAGTTGGAGGCATCTATA-3ʹ; siGYS2 #2, 5ʹ-GGATCTTTAACTGCCTGGTTCTTAA −3ʹ. Transfection was performed by the Lipofectamine 3000 according to the indicated manufacturer’s protocols. After 48 hours of transfection, the efficiency of GYS2 overexpression and silence was confirmed by qPCR and Western blotting, and the cells were collected for further functional analysis.

### RNA extraction and qPCR

According to the manufacturer’s instructions, a total RNA was isolated using Trizol reagent (Invitrogen, Carlsbad, CA, USA), and then the first strand cDNA was synthesized with reverse transcription system kit (Takara, Dalian, China). The calculation of the relative expression of GYS2 was referenced by β-actin. The primer sequences were as follows: GYS2 forward (F): 5ʹ-TGGTAAATATGTCGTTGCCCAA-3ʹ, GYS2 reverse (R), 5ʹ-GTAGTGTAGCGTGGGTTGTAAAT-3ʹ; β-actin forward (F): 5ʹ-GGGAAATCGTGCGTGACATTAAG-3ʹ, β-actin reverse (R): 5ʹ-TGTGTTGGCGTACAGGTCTTTG-3ʹ. The relative levels of gene expression were represented as ΔCt = Ct gene − Ct reference, and then the fold change of gene expression was calculated by a 2 ^−ΔΔCt^ method. The qPCR reactions were performed in triplicate.

### Western blot analysis

The procedure for Western blotting was as follows: the total protein was first extracted from the cell using a Protease inhibitor and a phosphatase inhibitor (Beyotime Biotechnology) and the concentration of the protein was determined by a BCA assay. The total proteins were extracted from the cells using protease inhibitor and phosphatase inhibitors (Beyotime Biotechnology) and centrifuged for 20 minutes at 12,000 g/min. The supernatant was collected and the concentration of the proteins was determined by BCA assay (Beyotime Biotechnology). Then, after electrophoresis and membrane transfer, the protein on PVDF membrane was incubated with the first antibody of GYS2 (1:500, Sigma), P53 (1:1000, Cell Signaling Technology), P21(1:1000, Cell Signaling Technology), and Cyclin D1(1:1000, Cell Signaling Technology) overnight at 4°C for 1 hour at room temperature and β-Actin (1:5000, cell signaling technology) was used as reference. The membrane was washed 3 times with TBST, and then incubated with secondary antibody at room temperature for 1 hour. The obtained bands used ECL to visualize and quantify using Gel-Pro analyzer.

### Cell proliferation assay

Cell Counting Kit-8 (Boster, Wuhan, China) was used to assess cell proliferation, according to the manufacturer’s protocol. In short, a total of approximately 5 × 10^3^ ICC cells were inoculated into a 96-well plate, treated with a 10 ml/well Cell Counting Kit-8 solution for an indicated period of time, and 450 nm absorbance values were measured at each time point and curves were plotted. All the experiments were carried out in triplicate.

### Colony formation assay

We evaluated the proliferation of ICC cells by colony forming assay, as follows: a total of about 1 × 10^3^ ICC cells were seeded into a 6-well plate and maintained at 37°C in DMEM medium containing 10% FBS. After about 2 weeks, the medium was discarded, fixed with 4% paraformaldehyde and stained with 0.1% crystal violet, finally calculated the number of colonies. All experiments were performed in triplicate.

### EdU assay

For EdU detection, first, pre-culture each group of cells with diluted EdU medium for 3 hours according to the instructions of mixture reagent kit (Keygene Biotech). Then, the cells were washed twice in PBS, then fixed in 4% paraformaldehyde for 30 minutes, infiltrated with 0.1% TritonX-100 at room temperature for 10 minutes, washed with PBS, added 100ul Apollo staining solution to each well cells, incubated in dark, room temperature and shaking table for 30 minutes, and finally stained the nucleus. DAPI solution was applied and confocal microscope was used to capture images immediately after dyeing.

### Cell migration assay

We evaluated cell motion capabilities through cell migration. Briefly described below: The cells (1 × 10^5^ per 200 μL medium) were inoculated into the upper layer of the transwell chamber (8-mm aperture, Millipore) containing serum-free medium, and the lower layer of the chamber was added with an appropriate amount of fresh medium containing 10% FBS. After incubation for 48 hours, the cells penetrating the basement membrane were fixed with 4% paraformaldehyde and stained with 0.1% crystal violet respectively, and finally counted under the microscope. The experiments were performed in triplicate.

### Cell invasion assay

The invasiveness of the cells was tested by the cell invasion experiment. According to the manufacturer’s instructions, the BD Matrigel (BD Biosciences) was diluted with a serum-free cell culture medium at a ratio of 1:8 at 4°C, 100 ml was uniformly applied to the upper surface of the chamber, and then 500 ul medium containing 10% FBS was added to the lower chamber, then the cells (2 × 10^5^ per 200 μL medium) were inoculated in the upper chamber. After 48 hours of culture, the cells on the surface of the upper chamber were wiped off, and then the cells penetrating to the bottom of the membrane were fixed in 4% paraformaldehyde and stained with 0.1% crystal violet. Finally, the cells were observed and calculated under a high-power microscope. The experiments were performed in triplicate.

### Statistical analysis

All statistical analyses were conducted with SPSS 19.0 software (SPSS, Chicago, IL, USA). We mainly used GraphPad Prism 8.0 (La Jolla, CA, USA) for image editing. Data are expressed as mean ± SD. According to the data, the following corresponding test methods are selected: Student’s t test, Fisher’s exact test and Chi-square test to analyze the significant differences between the groups. The overall survival (OS and DFS) was analyzed and plotted using Kaplan-Meier and log-rank test. P-value < 0.05 was defined as statistically significant.

## Results

In this work, based on the results of an integrated pan-cancer analysis of GYS2 in GEPIA database strongly suggested that the gene was significantly down-regulated in a variety of tumors, including cholangiocarcinoma. Therefore, we speculate that GYS2 may play an important role in the progress of ICC. Subsequently, we confirmed that the gene was down-regulated in ICC. Survival analysis showed that low GYS2 expression was associated with poor prognosis and was an independent risk factor for ICC patients. Further functional studies demonstrated that low expression of GYS2 could promote the proliferation, replication and invasiveness of ICC cells, which seems to be influenced by the classical P53 pathway.

### The expression of Glycogen Synthase 2 is markedly down-regulated in ICC

**Figure 1.**
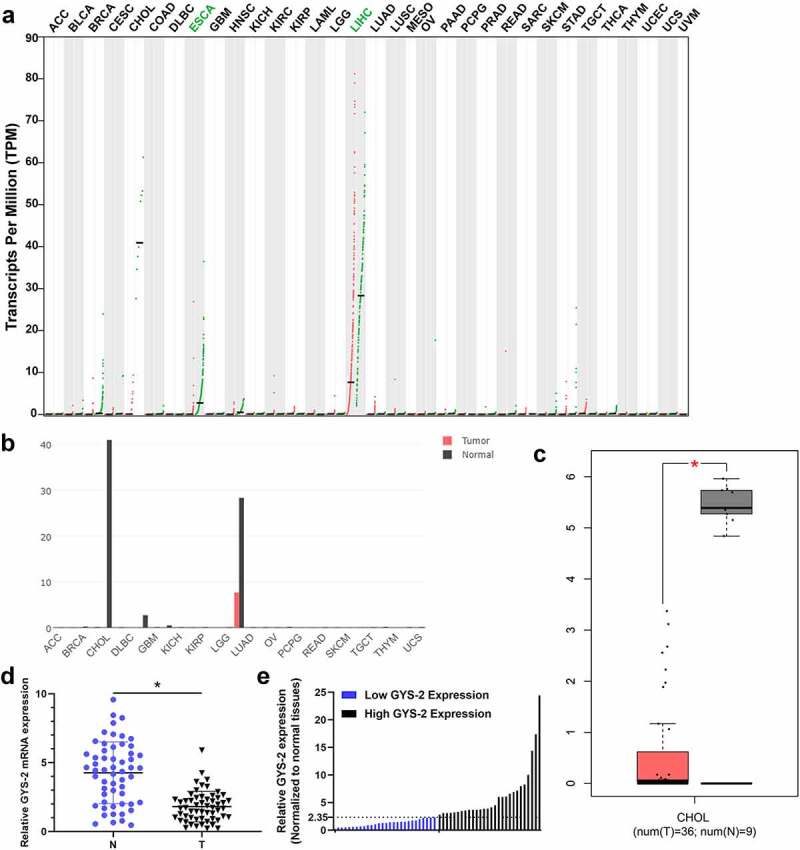
**The expression of Glycogen Synthase 2 is markedly down-regulated in ICC**. (a) An integrated pan-cancer analysis of GYS2 in GEPIA database, the green dots in the figure represented the expression level of normal tissues, the red dots represented the expression level of cancer tissues, ‘T’ represented tumor tissues, and ‘N’ represented paired normal organization. Expression abundance is measured by normalized transcripts per million (TPM). (b) GYS2 pan-cancer expression analysis in the GEPIA database, bar graphs of gene expression profiles of all tumor samples and pairs of normal tissues, the height of bar indicates the median expression of a specific tumor type or normal tissue (log-normalized TPM). (c) Box plot of GYS2 expression in cholangiocarcinoma, the data comes from the GEPIA database (T = 36, N = 9). (d) The GYS2 expression level was detected by qPCR in ICC patient tumor and adjacent non-tumor tissues (N = 56). The experiments were repeated 3 times. (e) According to the expression level of GYS2 in ICC tissues, high and low expression levels were stratified

First, we performed an integrated pan-cancer analysis of GYS2 in the GEPIA database and found that the expression of GYS2 was significantly down-regulated in a variety of human tumors (Figure 1(a)), especially in hepatobiliary tumors (Figure 1(b)), compared with corresponding normal tissues. Further extraction of the above data showed that GYS2 expression was also dramatically down-regulated in cholangiocarcinoma compared with adjacent normal tissues (Figure 1(c)). Based on the above bioinformatics analysis, we tentatively identified that GYS2 probably exist as a tumor suppressor gene in tumor progression. In order to further clarify the expression of GYS2 in ICC, a total of 56 pairs of matched cancers and adjacent non-cancerous tissues were collected, and the detailed clinicopathological indicators of these patients were shown in Table 1. Further qPCR analysis showed that the expression of GYS2 in ICC was markedly down-regulated compared with the corresponding adjacent non-cancerous tissues (Figure 1(d)). Further analysis of the relative expression of GYS2 in the above cancerous and adjacent non-cancerous tissues showed that the expression of GYS2 in the adjacent non-cancerous tissues of 56 patients was about 2.35 times of that in the paired tumor tissues. Therefore, taking 2.35 as the cutoff value, 56 patients with ICC could be divided into high expression group and low expression group on average (Figure 1(e)). Next, we aimed to investigate whether a low level of GYS2 was linked with clinicopathological characteristics in ICC patients. As shown in Table 2, the expression level of GYS2 was closely related to the pathologic differentiation (P = 0.017), tumor size (P = 0.026), vascular invasion (P = 0.015) and lymphatic metastasis (P = 0.014). These results indicated that dysregulated expression of GYS2 is involved in the progress of ICC.

**Table 1.** Baseline characteristics of the patients

Variable	Number	Percent (%)
Age (year)		
≤55	22	39.3%
>55	34	60.7%
**Pathologic differentiation**		
well	8	14.2%
moderately poorly	33 15	59.0% 26.8%
**Gender**		
male	32	57.1%
female	24	42.9%
**CA-199**		
≤35	10	17.9%
>35 **Tumor number** single multiple **Tumor size (cm)** ≤5 >5 **Vascular invasion** yes no	46 7 49 21 35 28 28	82.1% 12.5% 87.5% 37.5% 62.5% 50.0% 50.0%
**T stage**		
T1(1a&1b)	22	39.3%
T2 T3(3a&3b)	26 8	46.4% 14.3%
**N stage**		
negative	41	73.2%
positive	15	26.8%
**TNM stage (AJCC) ^a^**		
I(IA&IB)	22	39.3%
II III(IIIA&IIIB)	12 22	21.4% 39.3%

a: American Joint Committee on Cancer (AJCC), patients were staged in accordance with the 8th Edition of the AJCC Cancer’s TNM Classification.

**Table 2.** Relationship between GYS2 expression and clinicopathological parameters

Variable	High expression of GYS2	Low expression of GYS2	*P* value
Age (year)			0.272
≤55	9	13	
>55	19	15	
**Pathologic subtype**			0.017*
well	6	2	
moderately poorly	19 3	14 12	
**Gender**			0.412
male	13	19	
female	15	9	
**CA-199**			0.728
≤35	6	4	
>35 **Tumor number** single multiple **Tumor size (cm)** ≤5 >5 **Vascular invasion** yes no	22 23 5 15 13 9 19	24 26 2 6 22 19 9	0.421 0.026* 0.015*
**T stage**			0.252
T1(1a&1b)	14	8	
T2 T3(3a&3b)	11 3	15 5	
**N stage**			0.014*
negative	25	16	
positive	3	12	
**TNM stage (AJCC) ^a^**			0.087
I(IA&IB)	15	7	
II III(IIIA&IIIB)	5 8	7 14	

*Chi-square(and Fisher’s exact) test, P value<0.05.

a: American Joint Committee on Cancer (AJCC), patients were staged in accordance with the 8th Edition of the AJCC Cancer’s TNM Classification.

### Dysregulated Glycogen Synthase 2 was closely related to unfavorable prognosis of patients with ICC

**Figure 2.**
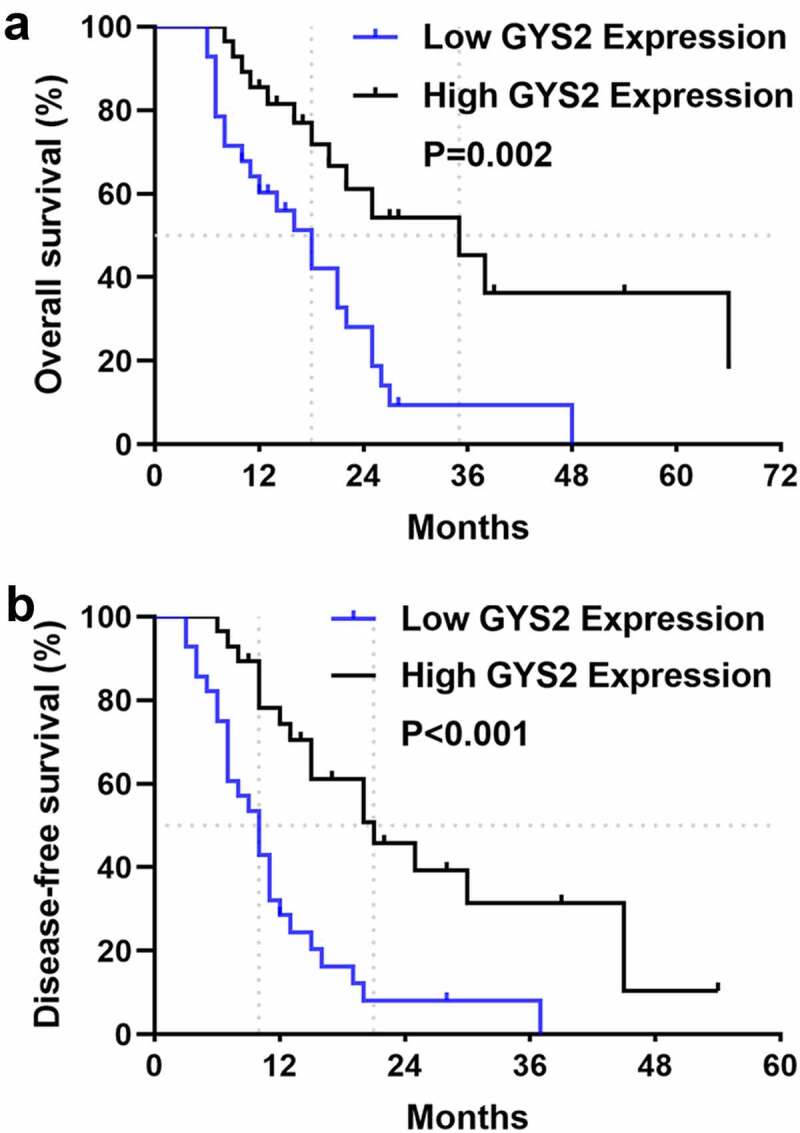
**Dysregulated Glycogen Synthase 2 was closely related to unfavorable prognosis of patients with ICC**. (a) Kaplan-Meier survival analysis showed that patients in the GYS2 low-expression group had worse overall survival (OS) than those in the high-expression group (P = 0.02). (b) Kaplan-Meier survival analysis showed that patients in the GYS2 low-expression group had shorter disease-free survival (DFS) than those in the high-expression group (P < 0.001)

In order to determine the prognostic significance of GYS2 expression for ICC patients, we attempted to relate the expression level of GYS2 to the clinical outcomes. In the survival analysis, 56 ICC patients with complete and reliable follow-up data were included. As shown in Figure 2(a), the median overall survival (OS) was 18 months in patients with low GYS2 expression and 35 months in patients with high GYS2 expression, suggesting a worse survival outcome in patients with low GYS2 expression. Similarly, as shown in Figure 2(b), patients with low GYS2 expression had significantly shorter disease-free survival (DFS) than those with high expression, suggesting a greater risk of early recurrence in patients with low GYS2 expression. Further, we conducted Cox regression model (proportional hazards regression model) to analyze the effects of various clinical indicators on overall survival (OS) and disease-free survival (DFS) of ICC patients. The results showed that the low expression of GYS2 was an independent risk factor for early recurrence and poor prognosis (refer to Table 3 and 4 in the supplementary materials for details). The above data highlighted that for ICC patients, low level of GYS2 expression indicates poor survival prognosis, and the predictive value of GYS2 makes it possible to become a valuable clinical prognostic biomarker.

### The expression of Glycogen Synthase 2 in ICC cell lines

**Figure 3.**
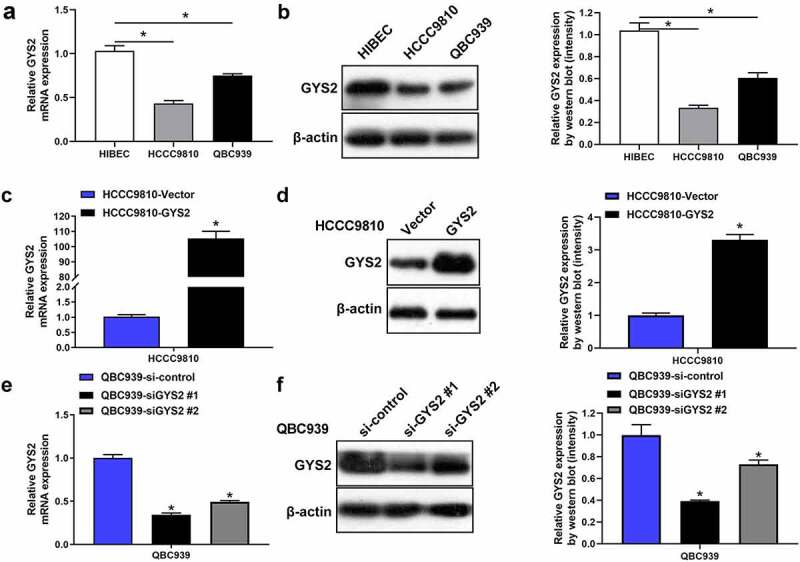
**The expression of Glycogen Synthase 2 in ICC cell lines**. (a) Relative mRNA and (b) protein expression levels of GYS2 in ICC cell lines (HCCC9810 and QBC939) and human normal intrahepatic bile duct epithelial cells (HIBECs). *P < 0.05. (c) The mRNA and protein expression levels (d) of GYS2 were detected by qPCR and Western blotting respectively, when GYS2 was exogenously overexpressed by transfecting pcDNA3.1 plasmid. (e and f) After GYS2 was silenced, the mRNA and protein expression of QBC939 cell line were detected by qPCR and Western blotting, respectively. *P < 0.05. All experiments were repeated 3 times

In order to further explore the biological function of GYS2 in ICC cells (HCCC9810 and QBC939), we identified the mRNA and protein expression levels of GYS2 in ICC cell lines by qPCR and Western blotting, respectively. The results showed that compared with human normal intrahepatic bile duct epithelial cells (HIBECs), the mRNA (Figure 3(a)) and protein expression levels (Figure 3(b)) of GYS2 were relatively lower in ICC cell lines. Furthermore, in view of the relatively low expression of GYS2 in HCCC9810 cell line and the relatively high expression in QBC939 cell line, we overexpressed GYS2 in HCCC9810 (Figure 3(c-d)) and silenced GYS2 expression in QBC939 cell line (Figure 3(e-f)), the expression levels of GYS2 were then detected by qPCR and Western blotting. After confirming the success of transfection, the cell line was used for subsequent cellular functional research.

### The effect of Glycogen Synthase 2 expression level on the proliferation of ICC cells in vitro

**Figure 4.**
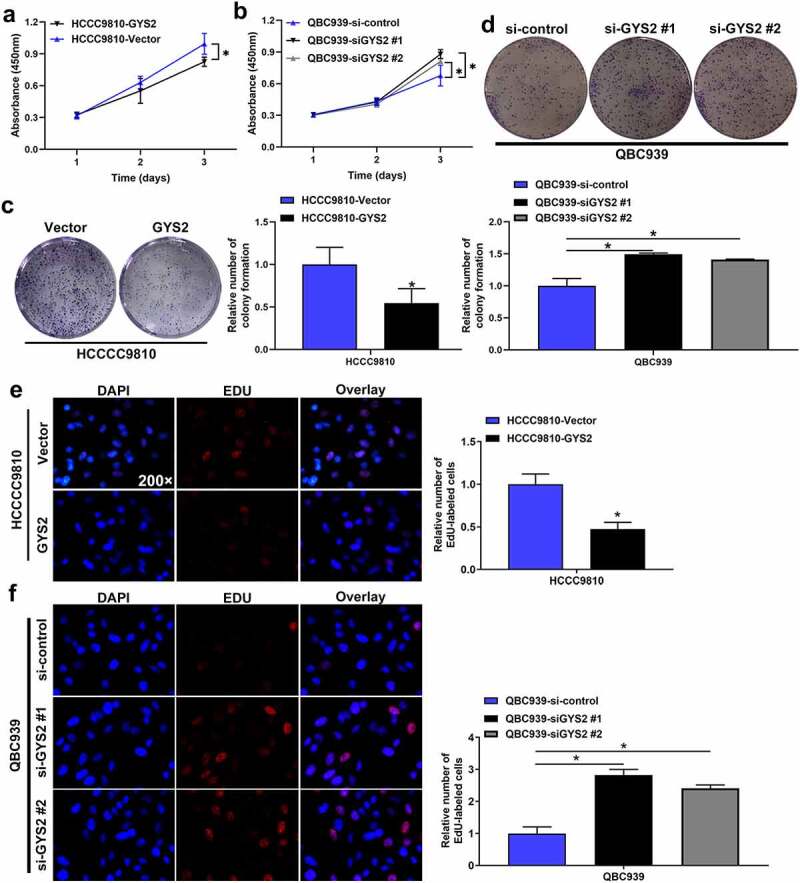
**The effect of Glycogen Synthase 2 expression level on the proliferation of ICC cells in vitro**. (a) Cell-counting kit-8 (CCK-8) assays demonstrated that the proliferation of HCCC9810 cells was significantly inhibited after endogenous overexpression of GYS2. (b) Cell-counting kit-8 (CCK-8) assays demonstrated that the proliferation of QBC939 cells was dramatically enhanced after silencing GYS2. (c) Colony formation assays showed that the colony forming ability of HCCC9810 cells was significantly impaired after endogenous overexpression of GYS2. (d) Colony formation assays demonstrated that the colony forming ability of QBC939 cells was prominently improved after silencing GYS2. (e) EdU assays indicated that the replication of DNA in HCCC9810 cells was significantly inhibited after endogenous overexpression of GYS2. (f) EdU assays indicated that the replication of DNA in QBC939 cells was prominently improved after silencing GYS2. *P < 0.05. All experiments were repeated 3 times

Cell-counting kit-8 (CCK-8) assays (Figure 4(a-b)), colony formation assays (Figure 4(c-d)) and EdU assays (Figure 4(e-f)) all demonstrated that the ability of proliferation and replication of HCCC9810 cells was significantly inhibited after endogenous over-expression of GYS2, whereas the above ability of QBC939 cells was visibly enhanced after silencing of GYS2.

### The effect of Glycogen Synthase 2 expression level on migration and invasion of ICC in vitro

**Figure 5.**
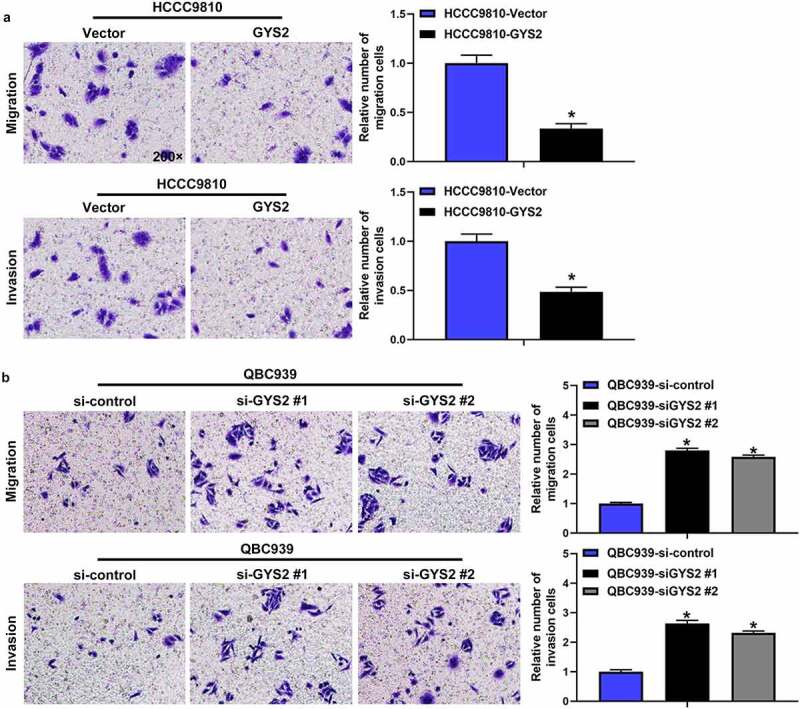
**The effect of Glycogen Synthase 2 expression level on migration and invasion of ICC in vitro**. (a) Overexpression of GYS2 significantly impaired the ability of migration and invasion in HCCC9810 cells. (b) After silencing GYS2, its migration and invasion ability was dramatically enhanced. *P < 0.05. All experiments were repeated 3 times

The ability of migration and invasion of tumor cells is essential for the spread and metastasis of tumors. Transwell assays demonstrated that endogenous overexpression of GYS2 dramatically impaired the migration and invasion of HCCC9810 cells (Figure 5(a)), while the ability of QBC939 cells was evidently enhanced after silencing of GYS2. (Figure 5(b)).

Collectively, these results suggested that GYS2 may play a role as a tumor suppressor gene in the progression of ICC. When GYS2 was overexpressed, the proliferation, migration and invasion of tumor cells were obviously limited, while when GYS2 was silenced, the reverse trend was observed.

### Glycogen Synthase 2 may exert antitumours effect via P53 signaling pathway

**Figure 6.**
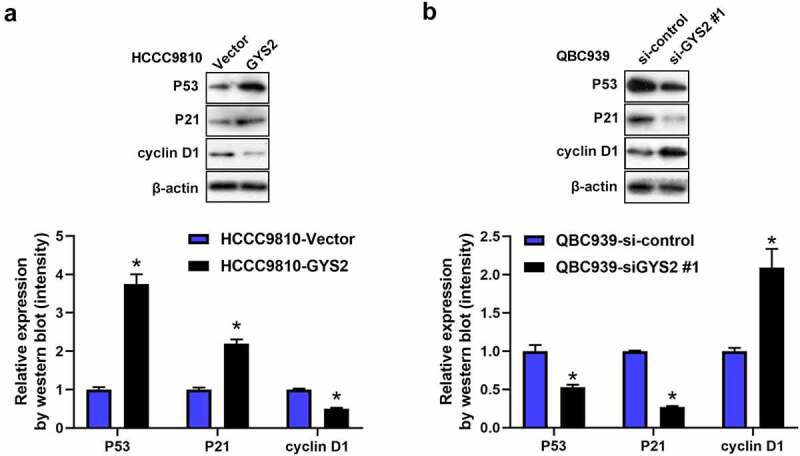
**Glycogen Synthase 2 may exert antitumours effect via P53 signaling pathway**. (a) After overexpression of GYS2, Western blotting was used to detect the changes in the protein expression levels of P53 and key genes (P21 and Cyclin D) downstream of the pathway. (b) After silencing GYS2, Western blotting was used to detect the changes in the protein expression levels of P53 and key genes (P21 and Cyclin D) downstream of the pathway. *P < 0.05. All experiments were repeated 3 times

Recently, it has been pointed out in the literature that GYS2 can participate in the progression of HCC by activating the P53 signaling pathway. In order to validate whether GYS2 exerted anti-tumor activities via P53 in ICC. Therefore, we conducted Western blotting assays. As shown in Figure 6(a-b), after overexpression of GYS2 in HCCC9810 cells, compared with the control cells, the protein expression levels of P53 and its downstream regulatory gene P21 were significantly up-regulated, while the protein expression level of cyclin D1 inhibited by P53 was markedly inhibited, interestingly, after silencing GYS2, it showed a completely opposite trend. Such a result prompted us that in terms of underlying mechanism, GYS2 was largely by activating the P53 signaling pathway to play an anti-tumor effect in ICC cells.

## Discussion

Intrahepatic cholangiocarcinoma was a heterogeneous and highly aggressive primary liver malignancy. In recent years, the incidence rate and mortality had increased year by year, however, it was frustrating that its treatment and survival prognosis were far less than HCC [15,16]. At present, ICC lacks sensitive tumor biological indicators and effective therapeutic targets [3]. Facing the dilemma of ICC, it is urgent for us to study the ICC molecular mutation and its potential mechanism on the basis of further understanding the ICC heterogeneity [17]. We had known that one of the most striking hallmark of tumors is reprogramming energy metabolism [6], such as the classic Warburg effect [18], in which there was a metabolic difference between tumors and normal tissues that produce large amounts of lactic acid through glycolysis, this effect could greatly promote cell proliferation and tumor growth [19]. Liver glycogen synthase encoded by GYS2 is the key rate limiting enzyme for glycogen synthesis [20]. Recent studies have shown that the down-regulation of GYS2 expression was significantly associated with poor prognosis in HCC [11]. However, we still have no knowledge about the role of GYS2 in ICC. In this study, for the first time, we found that the expression of GYS2 in ICC was also down-regulated, which was closely associated with the poor prognosis of patients. Meanwhile, we also conducted some work on the biological function and potential mechanism of this gene.

We first performed an integrated pan-cancer analysis of GYS2 in GEPIA database, and found that GYS2 was significantly down-regulated in a variety of tumors, especially in hepatobiliary tumors. Subsequently, we detected the expression of GYS2 in matched ICC clinical specimens by qPCR, and found that the GYS2 in ICC was observably down-regulated, which was consistent with the results of the previous bioinformatics analysis. More importantly, through the comprehensive analysis of clinicopathological indicators and follow-up information, we found that the expression level of GYS2 was closely related to pathological grade, tumor size, microvascular invasion and lymph node metastasis. Compared with the high expression group, the patients with low expression of GYS2 had higher risk of postoperative recurrence and worse long-term prognosis. The above conclusions suggested that GYS2 has a certain clinical prognostic value for ICC patients. After elucidating the clinical significance of GYS2, functional studies have shown that the proliferation, migration and metastasis of ICC cells are significantly impaired when GYS2 is overexpressed, and when GYS2 is silenced, the above-mentioned capabilities are dramatically improved, which fully demonstrates the GYS2 played a role of tumor suppressor gene in the progress of ICC.

It was well known that the P53 gene is the gene most associated with human tumors, and mutations in this gene occurred in more than 50% of almost all malignant tumors [21,22]. P53 is a tumor suppressor protein that regulates the expression of a variety of genes, including apoptosis, growth inhibition, and cell cycle progression, just like any other tumor suppressor, the P53 gene normally slows or monitors cell division [23]. Therefore, in terms of mechanism, given the above background and the anti-cancer nature of GYS2 [24,25], we suspected that GYS2 may exert its anti-cancer effect by activating P53 signaling pathway. As we hypothesized, when GYS2 was overexpressed or silenced, the protein expression level of P53 and the key genes downstream of the pathway changed correspondingly, which at least suggested that the P53 signal pathway was involved in the tumor suppressor process of GYS2. It should be pointed out here that further data collection was required to clarify the more detailed GYS2 regulation and metabolism mechanism.

To sum up, our work underlined the importance of GYS2 to the clinical value of ICC patients and the possibility of targeted therapy in the future.

## Conclusions

In conclusion, the present study has shown that low GYS2 expression is associated with an unfavorable clinical outcome of ICC, which is an independent risk factor affecting patient survival. GYS2 appears to influence ICC cell phenotype via the classical P53 pathway. These data, GYS2, are expected to be a prognostic marker for ICC patients and a potential target for drug intervention.

## Supplementary Material

Supplemental Material

## Highlights

- Low expression of Glycogen synthase 2 predicted unfavorable prognosis in intrahepatic cholangiocarcinoma patients.
- GYS2 overexpression can impair the activity of cholangiocarcinoma cells via P53.
- Glycogen synthase 2 can be a potential therapeutic target for ICC patients.
