# Supplementary material for: Value of glycogen synthase 2 in intrahepatic cholangiocarcinoma prognosis assessment and its influence on the activity of cancer cells
Source: Bioengineered. 2021 Dec 7;12(2):12167–78. doi: 10.1080/21655979.2021.2005224 (PMC8810034; doi:10.1080/21655979.2021.2005224)
Supplement: Supplemental Material [file KBIE_A_2005224_SM9867.zip › supplementary/Supplementary Tables.docx]

**Table 3** Univariate and multivariate analysis of Overall Survival (OS) in ICC patients (n=56)

| **Variables** | **Univariate analysis** | | |  | **Multivariate analysis** | | |
| --- | --- | --- | --- | --- | --- | --- | --- |
|  | **HR** | **95%CI** | **P-Value** |  | **HR** | **95%CI** | **P-Value** |
| **Gender** | 1.62 | 0.83-3.31 | 0.183 |  |  |  |  |
| **Age** | 1.02 | 0.98-1.05 | 0.322 |  |  |  |  |
| **Pathological (moderately or poor vs. well)** | 2.34 | 1.38-3.94 | 0.001^**^ |  | 1.74 | 0.82-3.68 | 0.149 |
| **CA-199** | 1.00 | 1.00-1.00 | 0.001^**^ |  | 1.00 | 1.00-1.00 | 0.094 |
| **Tumor number** | 2.56 | 1.51-6.79 | 0.005^**^ |  | 2.11 | 1.63-7.31 | 0.053 |
| **Tumor size** | 1.50 | 1.28-1.76 | 0.000^***^ |  | 1.33 | 1.07-1.66 | 0.011^*^ |
| **Vascular invasion** | 4.46 | 2.08-9.56 | 0.004^**^ |  | 0.99 | 0.31-3.17 | 0.990 |
| **GYS2 expression** | 3.00 | 1.47-6.11 | 0.002^**^ |  | 2.84 | 1.09-7.38 | 0.032^*^ |
| **TNM-T-Stage (T3 or T2 vs. T1)** | 2.18 | 1.48-3.22 | 0.003^**^ |  | 0.18 | 0.01-2.12 | 0.172 |
| **Lymphatic metastasis** | 6.32 | 2.84-14.03 | 0.000^***^ |  | 0.04 | 0.25-4.91 | 0.184 |
| **TNM-Stage (AJCC) (stage II or stage III vs. stage I)** | 2.25 | 1.66-3.06 | 0.001^**^ |  | 1.71 | 0.79-6.86 | 0.073 |

Abbreviations: HR hazard ratio, 95%CI 95% confidence interval, T stage tumor stage, TNM tumor node metastasis. Cox regression analysis, * p<0.05, ** p<0.01, *** p<0.001.

**Table 4** Univariate and multivariate analysis of Disease-free Survival (DFS) in ICC patients (n=56)

| **Variables** | **Univariate analysis** | | |  | **Multivariate analysis** | | |
| --- | --- | --- | --- | --- | --- | --- | --- |
|  | **HR** | **95%CI** | **P-Value** |  | **HR** | **95%CI** | **P-Value** |
| **Gender** | 1.78 | 0.93-3.40 | 0.079 |  |  |  |  |
| **Age** | 1.03 | 0.99-1.06 | 0.139 |  |  |  |  |
| **Pathological (moderately or poor vs. well)** | 0.58 | 0.37-0.90 | 0.015^*^ |  | 0.83 | 0.48-1.42 | 0.493 |
| **CA-199** | 1.00 | 1.00-1.00 | 0.021^*^ |  | 1.00 | 1.00-1.00 | 0.298 |
| **Tumor Number** | 2.12 | 1.37-6.42 | 0.012^*^ |  | 1.84 | 1.26-6.39 | 0.041^*^ |
| **Tumor Size** | 1.43 | 1.23-1.66 | 0.001^**^ |  | 1.37 | 1.10-1.71 | 0.004^**^ |
| **Vascular invasion** | 3.80 | 1.93-7.48 | 0.015^*^ |  | 2.60 | 1.02-6.64 | 0.046^*^ |
| **GYS2 expression** | 3.52 | 1.84-6.75 | 0.000^***^ |  | 4.87 | 2.32-10.22 | 0.002^**^ |
| **TNM-T-Stage (T3 or T2 vs. T1)** | 1.94 | 1.39-2.73 | 0.003^**^ |  | 1.00 | 0.29-3.41 | 0.999 |
| **Lymphatic metastasis** | 4.93 | 2.39-10.18 | 0.001^**^ |  | 3.42 | 0.63-9.32 | 0.222 |
| **TNM-Stage (AJCC) (stage II or stage III vs. stage I)** | 1.78 | 1.42-2.24 | 0.000^***^ |  | 1.02 | 0.42-2.64 | 0.969 |

Abbreviations: HR hazard ratio, 95%CI 95% confidence interval, T stage tumor stage, TNM tumor node metastasis. Cox regression analysis, * p<0.05, ** p<0.01, *** p<0.001.
