# Supplementary figures and images for: Value of glycogen synthase 2 in intrahepatic cholangiocarcinoma prognosis assessment and its influence on the activity of cancer cells
Source: Bioengineered. 2021 Dec 7;12(2):12167–78. doi: 10.1080/21655979.2021.2005224 (PMC8810034; doi:10.1080/21655979.2021.2005224)

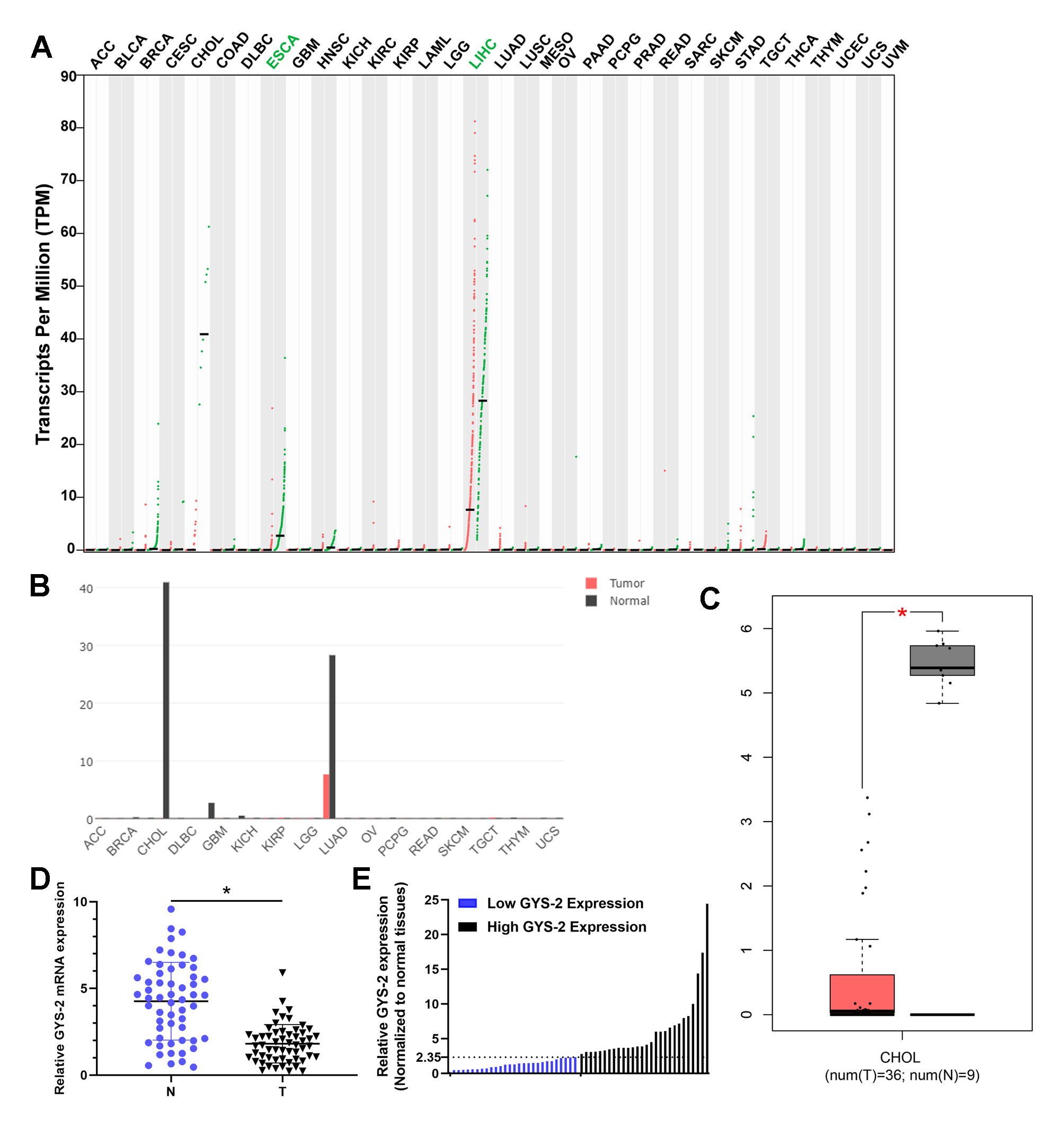

Supplement: Supplemental Material [file KBIE_A_2005224_SM9867.zip › supplementary/Supplementary Figure 1 (3).tif]
